# The effect of breast shielding during lumbar spine radiography

**DOI:** 10.2478/raon-2013-0004

**Published:** 2013-02-01

**Authors:** Nejc Mekis, Dejan Zontar, Damijan Skrk

**Affiliations:** 1 University of Ljubljana, Faculty of Health Sciences, Medical Imaging and Radiotherapy Department Ljubljana, Slovenia; 2 Slovenian Radiation Protection Administration Ljubljana, Slovenia

**Keywords:** breast dose, lead shielding, scattered radiation, lumbar spine radiography

## Abstract

**Background:**

The aim of the study was to determine the influence of lead shielding on the dose to female breasts in conventional x-ray lumbar spine imaging. The correlation between the body mass index and the dose received by the breast was also investigated.

**Materials and methods:**

Breast surface dose was measured by thermoluminescent dosimeters (TLD). In the first phase measurements of breast dose with and without shielding from lumbar spine imaging in two projections were conducted on an anthropomorphic phantom. In the second stage measurements were performed on 100 female patients, randomly divided into two groups of 50, with breast shielding only used in one group.

**Results:**

On average, breast exposure dose in lumbar spine imaging in both projections (anteroposterior (AP) and lateral) was found reduced by approximately 80% (p < 0,001) when shielding with 0.5 mm lead equivalent was used (from 0.45±0.25 mGy to 0.09±0.07 mGy on the right and from 0.26±0.14 mGy to 0.06±0.04 mGy on the left breast). No correlation between the body mass index (BMI) and the breast surface radiation dose was observed.

**Conclusions:**

Although during the lumbar spine imaging breasts receive low-dose exposure even when shielding is not used, the dose can be reduced up to 80% by breast shielding with no influence on the image quality.

## Introduction

Protection of the most radiosensitive organs is recommended during radiography procedures as even low exposure to ionizing radiation can damage cellular material and consequently lead to cancer.^1^ The most radiosensitive organs with the highest tissue weighting factor (0.12) include breast, lungs, stomach, colon, and bone marrow. According to ICRP the quoted weighting factor for breast represents an average over both sexes and is thus larger for females, further increasing the importance of breast shielding. Additionally the weighing factor is higher for females of younger age.^1^

In lumbar spine imaging which is one of the examinations with the highest radiation dose in conventional radiography breasts are located in close proximity of the primary x-ray beam.^2^–^6^ As lead shielding results in reduction of dose to different superficial organs in many radiological procedures^7^–^12^, the influence of breast shielding on breast exposure was considered an interesting subject of investigation.

Fordham *et al.* investigated whether breast dose is reduced in abdominal fluoroscopic examinations when lead shielding is used. They determined that, on average, the use of shielding with 0.5 mm lead equivalent reduces breast dose by 50%.^7^ Beaconsfield *et al*. examined whether lead shielding of breast during CT imaging of the head reduces exposure of the thyroid and breast. The measurements were performed both on a phantom and on patients. Results of the measurements on the phantom showed that the average of 0.27 mGy for unshielded breast was reduced by 90% when the shield was used. Measurements on the patients showed a reduction from on average 0.32±0.038 mGy to 0.075±0.042 mGy (approximately 70% reduction).^8^

Brnić *et al*. and Beaconsfield *et al*. also investigated the dose reduction to the breast in computed tomography (CT) of the head.^8^,^9^ They determined that using lead shielding of 0.35 mm lead equivalent reduced the dose to the breast by 57%, from on average 0.28±0.07 mGy to 0.13±0.05 mGy.^9^

Clancy *et al*. examined how different placing of the lead shield influences the dose to the gonads from lumbar spine imaging in AP and lateral positions. The measurements on a phantom were performed with and without the lead shield in the following positions: lead shield between the primary beam and phantom, lead shield between the phantom and the panel detector, and lead shield wrapped around the part of the phantom where gonads are located. Shielding with 0.4 mm lead equivalent was used. In the AP projection the dose to the testicles with a lead shield placed between the testicles and the x-ray tube was reduced by 42% (p ≤ 0.01) compared to the dose received without the shield; with the lead shield wrapped around the pelvis, the dose was only reduced by 36% (p ≤ 0.01). In the lateral projection, the dose received by the testicles when using the lead shield, wrapped around the pelvis, was reduced by 12% (p ≤ 0.06), compared to the dose received when the shield was not used.^12^

Review of the literature indicates that a significant dose reduction (over 50%) to the breast can be expected in the lumbar spine imaging by shielding the breasts. The aim of this research was to confirm this assumption and to determine the actual value of the dose reduction. Correlation of the breast dose with the body mass index (BMI) was also investigated using the linear regression and Pearson correlation coefficients.

## Materials and methods

The study was conducted in two phases. In the first phase measurements were conducted on an anthropomorphic phantom and in the second phase on 100 female patients, randomly divided into two groups of equal size, with breast shielding only used in one group. In both phases, breast dose was estimated from measurements of the surface dose.

In both phases measurements were carried at the Radiology department at Department of Orthopedic Surgery, University medical centre Ljubljana on the AXIOM Iconos R200 system with digital fluoroscopy, manufactured by Siemens. The grid ratio used was 17:1, with 70 line pairs/cm. The focus-detector distance (FDD) was 115 cm. The image detectors used were computed radiography (CR) imaging plates, AGFA CR 35-X (manufactured by Agfa-Gevaert N.V., Belgium) and MD 4.0 image plate’s size 35 × 43 cm.

Beam positioning was done as referred in the literature.^4^–^6^ In the AP projection, the transverse line of the central ray was positioned at the height of the lowest points of the rib cage, and the longitudinal on the central body line. In the lateral projection, the transverse line was at the same height whereas the longitudinal was in the frontal plane, 6 to 8 cm anterior from the posterior border of the skin of the back. In the lateral position the phantom and the patients were lying on the left side. The breasts were outside of the primary imaging field.

### Dosimetry

The entrance surface doses (ESD) were measured by LiBO_4_ thermolumnescent dosimeters (TLD). The TLDs were provided by and readings conducted at the Institute of Occupational Safety (Dosimetry Laboratory), one of the three approved dosimetry services in Slovenia. For each set of dosimeters five control dosimeters were used to record the background radiation, which was subtracted from the measurements. In addition the dose area product (DAP) measured with a built-in DAP meter (Kermax plus DDP, IBA Dosimetry) was also recorded.

### Measurements on phantom

**FIGURE 1.**
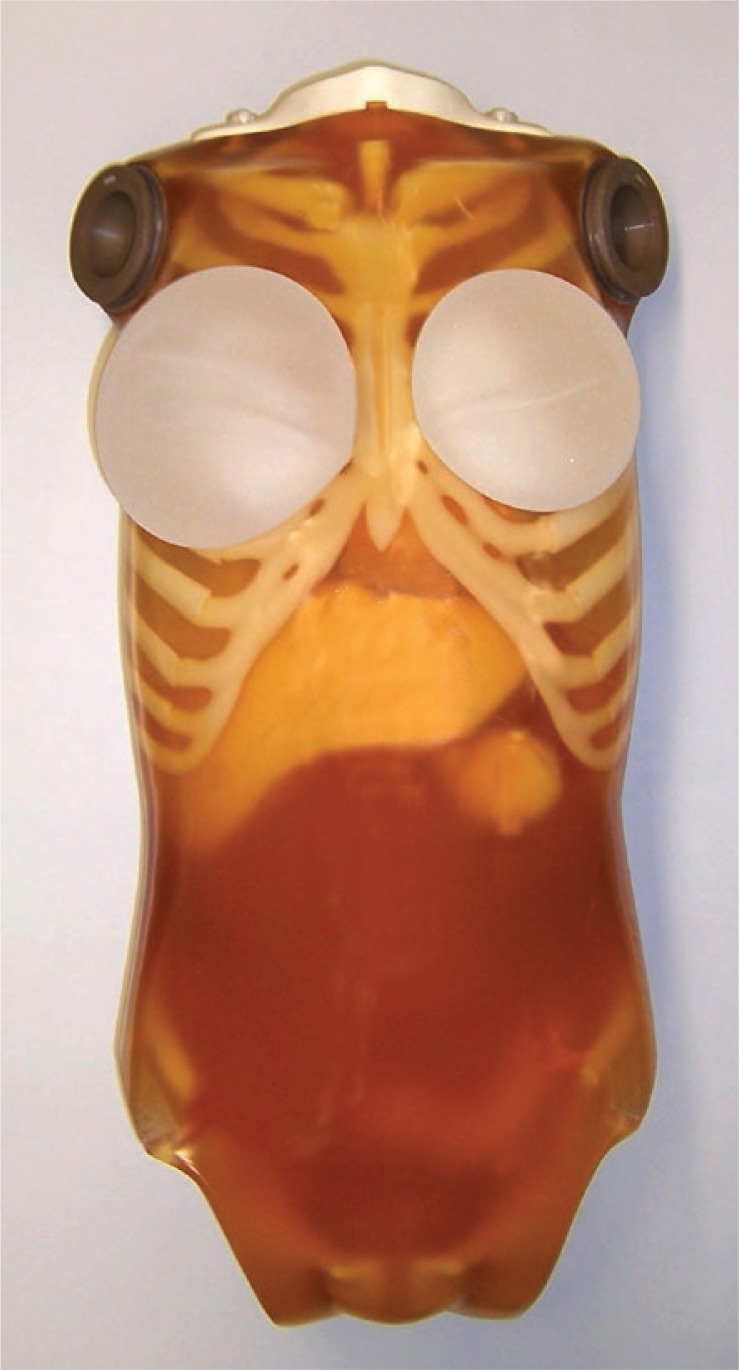
Image of the phantom with 340 ml implant size on the right and 500 ml implant size on the left.

**FIGURE 2.**
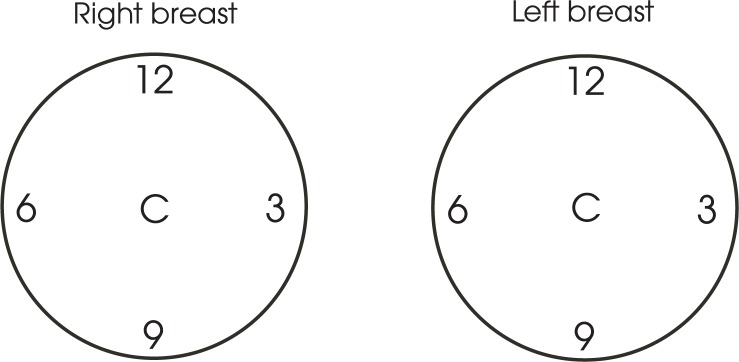
The position of the TLDs on the implants.

In the first part of the study the influence of breast shielding on their exposure was determined by measurements on an anthropomorphic phantom. A whole body phantom PBU 60 (Kyotokagaku Co., Ltd, Japan), simulating a man of 165 cm and 50 kg was used (Figure 1). The breasts were simulated by breast implants of two different sizes, 340 ml and 500 ml.^13^ The 340 ml size implants were positioned between 2^nd^ and 6^th^ rib and the medial edge was aligned with the edge of the sternum; the 500 ml size implants were positioned between 2^nd^ and 7^th^ rib with the medial edge aligned with the edge of the sternum, following the article from Marolt Mušič *et al*.^14^ TLDs were placed at the centre of the implant and on its edge as shown on Figure 2.

The phantom study was performed using the same protocol as used for lumbar spine radiography at this radiology department. After each positioning of the phantom, fluoroscopy was used to verify positioning of the lumbar spine. In the AP projection the tube voltage was 55 kV and in lateral projection 66 kV. The middle automatic exposure control (AEC) was used for both positions. Dose measurements were conducted for AP and lateral projection together so the results determine the breast dose for complete lumbar spine radiography.

### Measurements on patients

The second part of the study was conducted on 100 adult women (age range 35 – 89 years with average of 68.5 years) referred to lumbar spine radiography. The study was approved by the National Medical Ethics Committee. The patients were informed about the study and a written informed consent was obtained from all patients. None of the patients declined participation in the study.

The weight of the participating patients varied between 47 kg and 122 kg and their height between 150 cm and 183 cm. The patients were divided into two groups of 50 patients each. Kolmogorov-Smirnov test confirmed a natural distribution of variables BMI, DAP and breast dose in both groups (p was greater than 0.05 in all cases).

For each patient a single TLD was attached to the central part of each of the patients’ breasts. In the first group breasts were left unshielded as referred in the literature 3–6 and in the second group a shield of 0.5 mm lead equivalent was used to cover both breasts. The breast area was covered as tightly as possible with the shield remaining outside the primary beam.

As in the phantom study the locally used clinical protocol for the lumbar spine radiography was used. Thus, after positioning of the patient, fluoroscopy was used to examine the position of the lumbar spine. In the AP projection the tube voltage ranged from 55 kV to 75 kV and AEC was used. In the lateral projection the tube voltage ranged from 66 kV to 83 kV also with AEC. Again, the measurements show sum of exposure from both projections.

## Results

### Phantom study

In the phantom study 40 measurements with 1 TLD per measurement were performed. Tables 1 and 2 show absolute and percentile dose reduction due to the shielding for the 340 ml implant size and 500 ml implant size respectively. Positions of the numbers on the tables correspond to TLD positions on the implants as shown on Figure 1. In average the results of the phantom study show a dose reduction of approximately 80%.

### Patient study

In the second phase of the study, 200 measurements with TLD were carried out on 100 patients and for 94 patients DAP measurements were also recorded.

**FIGURE 3.**
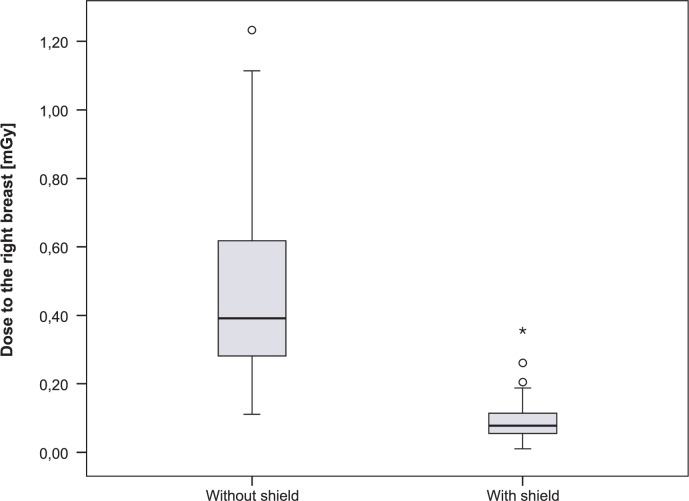
The comparison of the dose between shielded and unshielded right breast.

**FIGURE 4.**
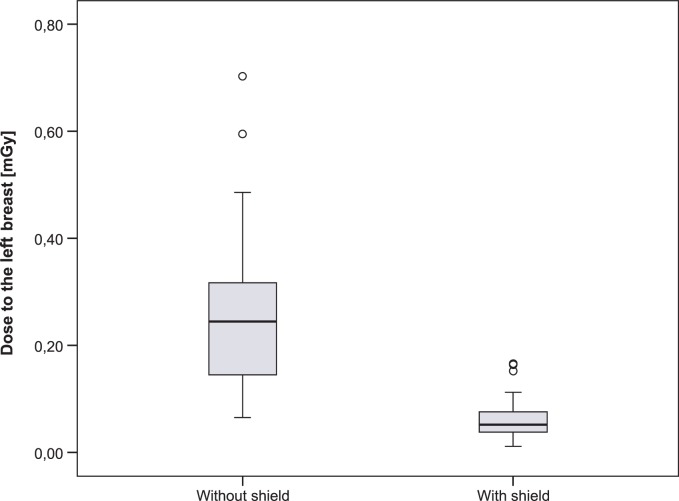
The comparison of the dose between shielded and unshielded left breast.

Results of the breast dose measurements are summarised in Tables 3 and 4. The results for both groups were analysed using two-sided t-test and statistically significant differences between the doses to the shielded and unshielded breast were confirmed with a significance of p < 0.05 (Figure 3, 4).

As a consequence of the lead shielding, the average dose for the right breast decreased from 0.45±0.25 mGy to 0.09±0.07 mGy, representing a statistically significant decrease confirmed by the two-sided t-test of independent samples (p < 0.001). The average dose value for the left breast decreased from 0.26±0.14 mGy to 0.06±0.04 mGy by using lead shield. The statistically significant decrease was confirmed by two-sided t-test of independent samples (p < 0.001). The difference between the dose to right and left breast was due to the patient’s positioning in the lateral projection. Patients were lying on their left side so the left breast was further away from the primary beam then the right.

In average, the breast surface radiation dose decreased by ∼80% when the lead shielding was used. Breast dose measurements (Table 3, 4) in both groups were analysed using two-sided t-test. Statistically significant differences between the doses to the shielded and unshielded breast were confirmed with a significance of p < 0.05 (Figures 3,4).

## Discussion

The results of the patient study were found to be consistent with the results of the phantom study as well as with observations of previous studies.^7^–^9^ They are showing a major (approximately 80%) reduction of the breast dose as a result of breast shielding.

In order to exclude the influence of other parameters in the patient study, eventual significant differences between the two groups of patients were checked for. The body mass index (BMI) and dose-area product (DAP) were identified as the main parameters that could influence the results. Distributions of those two parameters for both groups of patients were compared and checked for statistically significant differences. The two-sided t-test of independent samples was used to compare the body mass index (BMI) distributions and no statistically significant differences were found (p = 0.399). Despite differences in the size of the imaging field due to variations in physique among the patients, the two-sided t-test also showed no statistically significant differences (p = 0,195) in the DAP values between the two groups. It can thus be concluded that the observed differences in the breast doses can be attributed to the effect of the shielding.

In addition, the correlation between the breast dose and BMI was checked for. Using Pearson’s correlation coefficient, no linear correlation between BMI and dose to the breast was observed in either group (r = 0.211, p = 0.141; r = − 0.248, p = 0.082). These results are mostly consistent with findings of Brnić *et al*.^9^ who found no correlation between BMI and dose to the breast under the lead shield (c=0.28; p>0.05) and weak correlation between BMI and the breast without the lead shield (c=0.08; p>0.05).

The authors expect that further reduction of the breast doses could be achieved by using breast displacement device to remove the breasts further from the primary imaging field, as in the research conducted by Foley *et al*.^15^ Another option is the use of the PA projection instead of AP, as recommended by Brennan and Madigan^16^, and yet another alternative would be lead shielding, completely wrapped around the patient, as determined by Jackson and Brennan^11^, and Clancy *et al*.^12^ However, those approaches are much less practicable in regular clinical work and were not investigated in this study.

Various approaches such as optimisation of the imaging protocols and avoidance of fluoroscopy for verification of positioning could be implemented to achieve the breast dose reduction (and generally reduced patient exposure) during lumbar spine radiography. Although those might influence the relative effectiveness of shielding for breast protection (*e.g*. reduced shielding factor of lead at higher tube voltages), overall optimisation of the protocols was not the aim of this study.

## Conclusions

A breast dose reduction of approximately 80% (p < 0.001) during lumbar spine imaging was found to be achievable by using shield of 0.5 mm lead equivalent with no influence on the image quality. Despite a relatively low exposure of breast during this procedure, the use of breast shielding might thus be beneficial with little disturbance to established clinical practice. Although a higher dose reduction might be achieved by using displacement devices or full wrapping of lead shielding around the thorax, such measures are probably limited to academic interest due to their impracticality in clinical settings. No correlation between the BMI and the dose to the breast was observed.

## Figures and Tables

**TABLE 1.** Dose reduction on the phantom study with 340 ml implant size

	**Right breast (mGy)**			**Left breast (mGy)**	
	0.07 (− 73%)			0.02 (− 61%)	
0.16 (− 65%)	0.14 (− 84%)	0.16 (− 73%)	0.14 (− 71%)	0.10 (− 78%)	0.04 (− 59%)
	0.56 (− 67%)			0.81 (− 95%)	

**TABLE 2.** Dose reduction on the phantom study with 500 ml implant size

	**Right breast (mGy)**			**Left breast (mGy)**	
	0.11 (− 96%)			0.03 (− 86%)	
0.28 (− 86%)	0.16 (− 88%)	0.11 (− 64%)	0.32 (− 80%)	0.06 (− 80%)	0.06 (− 60%)
	0.65 (− 70%)			0.024 (− 65%)	

**TABLE 3.** Basic statistical characteristics of right breast dose measurement with and without the lead shielding

**Personal protective equipment**	**Average (mGy)**	**Median**	**Standard deviation**	**Minimum**	**Maximum**
Right breast without the lead shield	0.45	0.39	0.25	0.11	1.23
Right breast with the lead shield	0.09	0.08	0.07	0.01	0.36

**TABLE 4.** Basic statistical characteristics of left breast dose measurement with and without the lead shielding

**Personal protective equipment**	**Average (mGy)**	**Median**	**Standard deviation**	**Minimum**	**Maximum**
Left breast without the lead shield	0.26	0.24	0.14	0.07	0.70
Left breast with the lead shield	0.06	0.05	0.04	0.01	0.17
